# Supplementary material for: Equally Good Neurological, Growth, and Health Outcomes up to 6 Years of Age in Moderately Preterm Infants Who Received Exclusive vs. Fortified Breast Milk—A Longitudinal Cohort Study
Source: Nutrients. 2023 May 15;15(10):2318. doi: 10.3390/nu15102318 (PMC10223744; doi:10.3390/nu15102318)
Supplement: Supplementary file 1 [file nutrients-15-02318-s001.zip › nutrients-2384233-supplementary.pdf]

Table S1. Estimated marginal means (EMMs) compared to Swedish norm data, 95% confidence intervals (95% CIs) and p values in the respective feeding group and between the two feeding groups using linear mixed-effect models over SDSs and child weight, height, and head circumference. Adjustments were made for gestational age at birth, child sex, and maternal and paternal weight in the weight model. Adjustments were made for gestational age at birth, child sex, and maternal and paternal height in the height model and gestational age at birth and child sex in the head circumference model.

	Breast milk (n=43)	Fortified breast milk and/or formula (n=99)	Difference between the breast milk and fortified breast milk/formula groups	
	EMM (95% CI)	EMM (95% CI)	EMM (95% CI)	p value
**Weight**				
36 weeks of gestational age	-0.90 (-1.11--0.69)	-0.80 (-1.12--0.48)	-0.10 (-0.48-0.28)	0.61
40 weeks of gestational age	-0.87 (-1.07--0.66)	-0.78 (-1.10--0.45)	-0.09 (-0.47-0.30	0.65
6 months of age	-0.58 (-0.78--0.39)	-0.53 (-0.83--0.23)	-0.06 (-0.42-0.30)	0.75
1 year of age	-0.38 (-0.57--0.18)	-0.34 (-0.63--0.04)	-0.04 (-0.39-0.32)	0.84
1.5 years of age	-0.23 (-0.43--0.03)	-0.21 (-0.50-0.09)	-0.02 (-0.38-0.33)	0.89
2.5 years of age	-0.11 (-0.31-0.09)	-0.08 (-0.39-0.22)	-0.02 (-0.39-0.34)	0.90
4 years of age	-0.24 (-0.44-0.04)	-0.18 (-0.48-0.13)	-0.06 (-0.43-0.30)	0.74
5.5 years of age	-0.47 (-0.79--0.14)	-0.47 (-0.79--0.14)	-0.13 (-0.52-0.25)	0.50
**Height**				
36 weeks of gestational age	-0.30 (-0.50--0.09)	-0.34 (-0.65--0.02)	0.04 (.0.33-0.42)	0.83
40 weeks of gestational age	-0.30 (-0.50--0.10)	-0.34 (-0.65--0.02)	0.04 (-0.33-0.41)	0.83
6 months of age	-0.32 (-0.51--0.13)	-0.35 (-0.64--0.05)	0.02 (-0.37-0.90)	0.90
1 year of age	-0.35 (-0.53--0.17)	-0.35 (-0.64--0.08)	0.01 (-0.33-0.34)	0.97
1.5 years of age	-0.37 (-0.55--0.20)	-0.36 (-0.63--0-09)	-0.01 (-0.33-0.31)	0.95
2.5 years of age	-0.42 (-0.60--0.25)	-0.38 (-0.65--0.12)	-0.04 (-0.36-0.28)	0.79
4 years of age	-0.50 (-0.70--0.30)	-0.41 (-0.71--0.11)	-0.09 (-0.45-0.27)	0.62
5.5 years of age	-0.58 (-0.82--0.33)	-0.43 (-0.80--0.06)	-0.14 (0.59-0.31)	0.53
**Head circumference**				
36 weeks of gestational age	-0.18 (-1.55-1.20)	0.35 (-1.46-1.48)	0.52 (-1.07-2.12)	0.52
40 weeks of gestational age	-0.17 (-1.48-1.15)	0.34 (-0.43-1.11)	0.51 (-1.02-2.03)	0.51
6 months of age	-0.05 (-1.01-0.99)	0.28 (-0.38-0.94)	0.33 (-0.91-1.56)	0.60
1 year of age	0.07 (-1.38-1.52)	0.22 (-0.76-1.20)	0.15 (-1.60-1.90)	0.87
1.5 years of age	0.19 (-2.00-2.38)	0.16 (-1.31-1.63)	-0.03 (-2.67-2.61)	0.98
